# A new tool for prioritization of sequence variants from whole exome sequencing data

**DOI:** 10.1186/s13029-016-0056-8

**Published:** 2016-07-01

**Authors:** Brigitte Glanzmann, Hendri Herbst, Craig J. Kinnear, Marlo Möller, Junaid Gamieldien, Soraya Bardien

**Affiliations:** Division of Molecular Biology and Human Genetics, Faculty of Medicine and Health Sciences, Stellenbosch University, Cape Town, South Africa; Department of Law, Faculty of Law, Stellenbosch University, Cape Town, South Africa; SA MRC Centre for TB Research, DST/NRF Centre of Excellence for Biomedical TB Research, Division of Molecular Biology and Human Genetics, Faculty of Medicine and Health Sciences, Stellenbosch University, Cape Town, South Africa; South African National Bioinformatics Institute, University of the Western Cape, Cape Town, South Africa

**Keywords:** TAPER™, Whole exome sequencing, Bioinformatics capacity, Variant identification

## Abstract

**Background:**

Whole exome sequencing (WES) has provided a means for researchers to gain access to a highly enriched subset of the human genome in which to search for variants that are likely to be pathogenic and possibly provide important insights into disease mechanisms. In developing countries, bioinformatics capacity and expertise is severely limited and wet bench scientists are required to take on the challenging task of understanding and implementing the barrage of bioinformatics tools that are available to them.

**Results:**

We designed a novel method for the filtration of WES data called TAPER™ (Tool for Automated selection and Prioritization for Efficient Retrieval of sequence variants).

**Conclusions:**

TAPER™ implements a set of logical steps by which to prioritize candidate variants that could be associated with disease and this is aimed for implementation in biomedical laboratories with limited bioinformatics capacity. TAPER™ is free, can be setup on a Windows operating system (from Windows 7 and above) and does not require any programming knowledge. In summary, we have developed a freely available tool that simplifies variant prioritization from WES data in order to facilitate discovery of disease-causing genes.

**Electronic supplementary material:**

The online version of this article (doi:10.1186/s13029-016-0056-8) contains supplementary material, which is available to authorized users.

## Background

Rapid developments in high throughput sequence capture methods as well as in next generation sequencing (NGS) approaches have made whole exome sequencing (WES) both technically feasible and cost-effective. Moreover, the success of WES in the discovery of novel disease-causing mutations in numerous rare diseases is well established [1, 2]. WES can typically yield tens of thousands of variants per sequenced individual; the generation of data for analysis is therefore not considered to be the challenge with WES or NGS, but rather the way in which data is analysed is proving to be the major conundrum [3]. The identification of a single, plausible disease-causing mutation for a particular disease is proving to be as difficult as looking for the proverbial “needle in a haystack”. In addition to this, it has been well documented that every individual or pedigree will carry several so-called private mutations that do not cause overt disease [4]. Although numerous software tools are available that can aid in the prioritization of candidate disease-causing variants, all of the functionalities are disseminated in various analytical tools and researchers are forced to do all the analyses separately and then pool all of the results together. This task is both time-consuming and demands a considerable understanding of each of the bioinformatics tools that are used [3, 5]. Moreover, there are instances where different functional prediction tools provide inconsistent results [5] thereby making it exceptionally difficult to obtain a short list of candidates for validation and further follow up studies.

Additionally, in developing countries the limited number of bioinformaticists and the lack of adequate computational infrastructure further limit the successful application and implementation of NGS technologies. The paucity of trained bioinformaticists means that wet bench scientists with limited bioinformatics knowledge are left with the daunting task of prioritizing candidate disease-causing variants from files in a variant called format (VCF). Annotation of the data using programs such as SeattleSeq (http://snp.gs.washington.edu/SeattleSeqAnnotation141/) or ANNOVAR [6] from the VCF file focus on the comprehensive annotation of variants using information from assorted bioinformatics resources which include gene features, genomic conservation and possible pathogenicity [3].

Accordingly, to meet these challenges we developed TAPER™ (Tool for Automated selection and Prioritization for Efficient Retrieval of sequence variants), which encompasses a number of steps to prioritize sequence variants into one analytical procedure. TAPER™ has the potential to empower researchers in resource-constrained environments and should enable them to generate a short list of variants for further analyses. Moreover, we aim to do this using a hypothesis-free approach whereby all information (mathematical and statistical) is taken into account for novel variant identification. TAPER™ can be used even when the details of the phenotype, affection status and the inheritance pattern are unclear. Additionally, we evaluated the performance of TAPER™ through the use of a number of proof-of-concept examples using four WES datasets containing known causal mutations for three Mendelian disorders. And finally, we compared TAPER to freely available bioinformatics filtration pipelines namely PhenIX [7] and Exomiser [8].

## Implementation

**Fig. 1:**
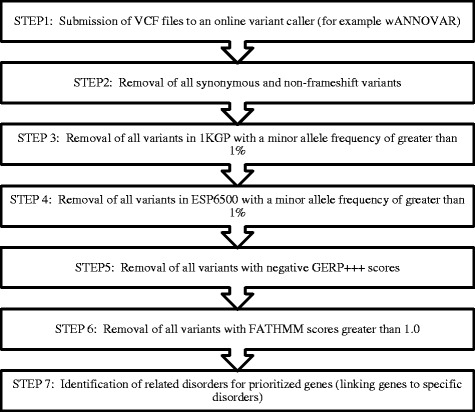
The seven-level filtration framework that makes up the backbone of TAPER™. (Abbreviations: 1KGP – 1000 Genomes Project; ESP6500 – Exome Sequencing Project 6500; GERP – Genomic Evolutionary Rate Prediction Score; FATHMM – Functional Annotation Through Hidden Markov Models)

TAPER™ is composed of a number of steps to filter and prioritize candidate variants (single nucleotide variants (SNVs) and indels) across individual patients that have been subjected to WES. TAPER™ was constructed using Microsoft Visual Studio Professional 2013 (Microsoft Corporation, Microsoft Redmond Campus, Redmond, Washington, United States) and additional packages downloaded in order to support the development of TAPER™ included Visual C#, CSV Helper (http://joshclose.github.io/CsvHelper/) and HTML Agility Pack (https://htmlagilitypack.codeplex.com/). It should be noted that TAPER™ has been designed in such a way as to filter all prioritized variants according to default settings. Alternatively, the user is able to alter each parameter according to their specific needs. The preliminary step involves obtaining the raw, unaligned sequences in FASTQ format. Quality control is then performed using FastQC (http://www.bioinformatics.babraham.ac.uk/projects/fastqc/) and aligned to the NCBI Human Reference Genome hg19 using NovoAlign v2 (http://www.novocraft.com/main/page.php?s=novoalign). The outcome of the alignment file is a VCF file, which is generated using the Mpileup function in SAMTools (version 1.4). Submission of the aforementioned VCF file to wANNOVAR (http://wannovar.usc.edu/) generates a downloadable text document, which is then in turn, submitted to TAPER™. wANNOVAR is the online version of the ANNOVAR, which is more user friendly for those individuals who have limited or no command line experience. TAPER™ accommodates structured tab-delimited text files and basic text files. Each of these formats can contain annotation information as well as genotypes for numerous samples. The seven-level filtration framework for TAPER™ is illustrated in Fig. 1.

These seven steps can be explained as follows:

1. The submission of the processed.vcf files to an online variant caller such as wANNOVAR (http://wannovar.usc.edu/), allows for the annotation of functional consequences of genetic variants from high-throughput sequencing data. This procedure is performed through an independent submenu in TAPER™.
2. Removal of all synonymous variants as well as variants that do not cause frameshifts – synonymous variations are defined as codon substitutions that do not change the amino acid and are unlikely to be the underlying cause for rare diseases – for this reason these variants, along with those that do not cause frameshifts, are removed from the list of prioritized variants.
3. Removal of variants in the 1000 Genomes Project (1KGP) (http://www.1000genomes.org/) that are found at a frequency of greater than 1 % - any variant that is found in the 1KGP database at a frequency of 1 % or less is considered to be rare. It is hypothesized that rare variants are likely to cause disease and for this reason, variants with very low or no available frequency data are prioritized.
4. Removal of variants in the Exome Sequencing Project (ESP) 6500 (http://evs.gs.washington.edu/EVS/) with a frequency of greater than 1 % - this second frequency based step is based on that of the 1KGP data (STEP 3). Rare, possible disease-causing variants are likely to be at an extremely low frequency in this database and any variant with a frequency that is higher than 1 % is removed from the list of interest.
5. Removal of variants with negative GERP scores – Genomic Evolutionary Rate Profiling (GERP) scores are the conservation scores from dbNSFP (database for nonsynonymous SNPs functional predictions) (http://sites.google.com/site/jpopgen/dbNSFP; [9]; higher scores are indicative of greater conservation; scores of > 0 are considered to be conserved.
6. Removal all variants with positive FATHMM scores - functional analysis through hidden Markov Models (FATHMM) scores are used to determine the species-specific weightings for the predictions of the functional effects of protein missense variants [10]. The use of FATHMM scores have been shown to outperform the conventional prediction methods such as SIFT, PolyPhen2 and MutationTaster [10, 11]. Positive FATHMM scores predict a tolerance to the variation while negative FATHMM scores predict intolerance to the variation, and is subsequently considered to be pathogenic. Following proof of concept analysis it was determined that the best possible cut-off value for the FATHMM score is 1.0.
7. Identification of associated disorders for prioritized genes – the final step of TAPER™ determines whether the genes of interest have been linked to any other disorders using OMIM (Online Mendelian Inheritance in Man) (http://www.omim.org/) database as well as the DISEASES (http://diseases.jensenlab.org) database. If any of these disorders are related or similar to the disease of interest (e.g. both are brain disorders), then that gene and variant will become the main candidate for further analysis.

## Results

Proof of concept experiments were conducted in order to determine the efficacy of TAPER™. This was done on existing processed WES data for which the disease-causing gene had previously been identified using alternative methods. This was performed in order to determine whether or not the pipeline was effective as well as to determine specific cut-off values for each of the parameters used. The datasets that were used were sourced from various collaborators (Dr. Suzanne Lesage, Institut du Creveau et de la Moelle épinière, Hôpital Pitié Salpêtrière, Paris, France [12]; Mr Daniel J. Evans, Centre for Applied Neurogenetics, University of British Columbia, Vancouver, Canada) as well as files sourced from previously published papers; the datasets used were those that previously identified variants in Parkinson’s disease as well as severe intellectual disability and microcephaly and ataxia and myoclonic epilepsy [13, 14]. Through the use of these experiments, it was determined that the only filtration parameter that was too stringent was the FATHMM score. Adjusting the FATHMM score cut-off to 1.0 as opposed to removing all variants with a positive FATHMM score, allowed for the prioritisation of the four genes in each of the datasets. However, due to its known high discriminative power, we recommend the standard cut-off of less than -1.5 as the default TAPER™ starting point to ensure broad utility across a diversity of exome studies. TAPER™ was therefore successfully used to identify the same variants that had previously been implicated in specific diseases; *FBOX7* (L34R) and *PARK2* (R275W and M432V) in Parkinson’s Disease (PD); *SLC1A4* (E256K) in intellectual disability and microcephaly and *KCNA2* (R297Q) in ataxia and myoclonic epilepsy (Table 1). In addition, we compared the results obtained though TAPER™ when compared to two other open source variant prioritization tools (Additional file 1: Table S1). TAPER™ is a hypothesis-free software package so no prior information regarding a particular disease is necessary. For each of the other software packages used, a mode of inheritance as well as disease phenotypes are used to prioritize variants. This may generate a selection bias and for this reason, in small-scale laboratories where limited clinical information is available pertaining to the patients, candidate variants may be lost as a result of variant selection based on clinical features and inheritance patterns.

**Table 1:** Stepwise breakdown of results obtained by TAPER™ using WES datasets for which the causal mutations have previously been identified

	Parkinson’s disease dataset 1 –*FBOX7* (L34R) ^[12}^			Intellectual disability and microcephaly dataset 1 –*SLC1A4* (E256K) ^[13}^		Ataxia and myoclonic epilepsy dataset 1 – *KCNA2* (R297Q) ^[14}^	Parkinson’s disease dataset 2 - *PARK2* (R275W and M432V)		
	Individual_1	Individual_2	Individual_3	Individual_1	Individual_2	Individual_1	Individual_1	Individual_2	Individual_3
Total number of variants in VCF file	55 726	55 336	55 289	54 426	54 574	60 128	104 307	108 243	97 833
STEP 1: Total number of variants assigned to exonic regions by wANNOVAR	19 727	19 969	20 353	24 573	24 425	23 163	19 850	19 972	19 863
STEP 2: All synonymous and non-frameshifts removed	9 465	9 544	9 766	12 227	12 248	11 693	9 752	9 777	9 838
STEP 3: Remove all variants with a frequency >1 % in 1KGP	1 281	934	966	2 177	2 153	1 377	1 771	1 681	1 932
STEP 4: Remove all variants with a frequency >1 % in ESP6500	917	797	819	1 928	1 906	941	1 335	1 445	1 575
STEP 5: Remove all variants with negative GERP+++ scores	718	615	651	1 243	1 261	688	1 014	1 126	1 232
STEP 6: Remove all variants with FATHMM scores greater than 1.0	262	224	240	252	231	257	413	301	328
STEP 7: Variants linked to relevant diseases	252	221	236	241	221	239	398	262	292
Variant of interest in shortlist?	Yes	Yes	Yes	Yes	Yes	Yes	Yes	Yes	Yes

## Discussion

WES has provided a means for researchers to gain access to a highly enriched subset of the human genome in which to search for variants that are likely to be pathogenic and possibly provide important insights into a particular disease. TAPER™ was developed to support the need of biomedical researchers and bioinformaticists alike. The aim behind TAPER™ for the biomedical researcher is to provide an environment that can be used to visualize and interpret variation data that is obtained from WES sequencing platforms. In addition, TAPER™ differs significantly from other variant prioritization tools, as it does not require a hypothesis driven approach. This means that phenotypic information about the disease of interest as well as factors such as inheritance patterns and knowledge of pathways are not required to aid in variant prioritization. TAPER™ has applications in the broader sense When the outputs of TAPER™ are compared to those obtained from other software packages, TAPER™ generates a list of variants that is more manageable due to smaller numbers and thereby making variant selection for further analysis easier. This is illustrated when running the same datasets through other freely available software such as PhenIX and Exomiser. PhenIX ranks candidate variants based on variant pathogenicity as well as phenotypic similarity of diseases associated with genes harbouring these variants to the phenotypic profile of the individual. PhenIX generated an average of 632 variants per individual that was processed through this pipeline. Data processed through the Exomiser is prioritized based on variant frequency, inheritance patterns, phenotypic data, pathogenicity and quality. For each of the datasets that were processed through the Exomiser, an average of 816 candidate variants were obtained across the various individuals (Additional file 1: Table S1). Moreover, for bioinformaticists TAPER™ allows for VCF files that have been processed to be given to the biomedical researcher, thereby allowing the researcher to independently prioritize and interpret WES results. TAPER™ is able to read pre-annotated variation data from numerous file formats and allows users to search, sort and sift through larger data set, whether by a predetermined set of parameters or using each of TAPER™’s seven functions independently. The efficiency and accuracy of TAPER™ was tested using sets of data which contained known pathogenic mutations – this allowed for the identification of less stringent cut-off criteria which otherwise would have allowed for the loss of possible disease-causing variants. However, due to its known high discriminative power we use the recommended FATHMM cut-off score of less than 1.0 as a default starting point in TAPER™ to ensure broad utility and high predictive value. Overall, TAPER™ will allow groups of researchers, particularly those in resource constrained laboratories with very limited bioinformatics capacity and resources, to interpret and analyse sequence variation data, thereby making NGS technologies such as WES more feasible in these laboratory setups.

## Limitations of TAPER™

Given the general approach to WES data analysis through TAPER™, one of the major limitations is the fact that TAPER™ can only be used once a VCF file has already been generated. This result means that a biomedical researcher is still largely dependent on bioinformatics capacity in order to perform quality control and sequence alignment on samples that have been sequenced. This may prove to be a significant limitation for small-scale laboratories or in cases where bioinformatics capacity is restricted. In addition to this, another limitation of the TAPER™ program is the fact that cloud based storage is not yet possible. TAPER™ is not yet able to upload filtered results to data servers such as Dropbox (www.dropbox.com) or Google Drive (www.google.com/drive) – which may become problematic with large numbers of sample sets. Finally, TAPER™ can only be used on a Windows operating system, which is limiting to individuals who may use Linux or Macintosh (iOS) based systems.

## Future Work

TAPER™ has been designed in such a way that the software can later be amended to process different NGS data – this will include whole genome sequencing, transcriptome analysis and targeted resequencing. It is anticipated that we will include an additional filtration criteria, namely the use of the ExAC Genome database (http://exac.broadinstitute.org/) as this is the largest publically available database with exome sequencing data. In addition to this, TAPER™ will be modified to provide an interactive and easy means for SAM and BAM file conversions, conversion and merging of multiple BAM files as well as to provide a local, fast way to determine sequence coverage. We also intend to add an additional submenu that will allow the user to perform sequence manipulation – for example multiple sequence alignments, sequence assemblies and manipulation as well as BLAST requests. This will make TAPER™ a single software package that can be used for multiple purposes by biomedical researchers with limited bioinformatics capacity.

## Conclusion

NGS approaches such as WES have been able to provide significant insights into human disease. In scientific environments where computational capacity as well as resources are less restrictive, WES can provide much insight but concurrently generate a massive “computational headache”. Through the development of TAPER™, we have provided a single, easy to use, integrated approach to prioritize specific variants that could be linked to a specific disease. It is anticipated that as more researchers in developing countries move towards NGS and massively parallel sequencing, TAPER™ provides a simple, intuitive tool that may help highlight potential disease-causing variants.

## Additional files

Additional file 1:
**Table S1.** Results obtained from the comparison of TAPER™ to two other whole exome sequencing data analysis tools. (DOCX 18 kb)
