# Supplementary material for: A new tool for prioritization of sequence variants from whole exome sequencing data
Source: Source Code Biol Med. 2016 Jul 1;11:10. doi: 10.1186/s13029-016-0056-8 (PMC4929716; doi:10.1186/s13029-016-0056-8)
Supplement: Additional file 1: — Table S1. Results obtained from the comparison of TAPER™ to two other whole exome sequencing data analysis tools. (DOCX 18 kb) [file 13029_2016_56_MOESM1_ESM.docx]

Supplementary Table 1: Results obtained from the comparison of TAPER™ to two other whole exome sequencing data analysis tools

|  |  | **TAPER™** | **PhenIX** | **Exomiser** |
| --- | --- | --- | --- | --- |
| **Parkinson’s disease dataset 1 –*FBOX7* (L34R)** | Human Phenotype Ontology terms | None | Bradykinesia, resting tremor, postural instability, rigidity | Bradykinesia, resting tremor, postural instability, rigidity |
|  | Frequency cutoff | 0.01 | 0.01 | 0.01 |
|  | Mode of inheritance | None | Unknown | Unknown |
|  | **Average number of variants in final list*** | **34** | **626** | **813** |
| **Intellectual disability and microcephaly dataset 1 –*SLC1A4* (E256K)** | Human Phenotype Ontology terms | None | Bradykinesia, resting tremor, postural instability, rigidity | Bradykinesia, resting tremor, postural instability, rigidity |
|  | Frequency cutoff | 0.01 | 0.01 | 0.01 |
|  | Mode of inheritance | None | Unknown | Unknown |
|  | **Average number of variants in final list*** | **57** | **651** | **802** |
| **Ataxia and myoclonic epilepsy dataset 1 – *KCNA2* (R297Q)** | Human Phenotype Ontology terms | None | Bradykinesia, resting tremor, postural instability, rigidity | Bradykinesia, resting tremor, postural instability, rigidity |
|  | Frequency cutoff | 0.01 | 0.01 | 0.01 |
|  | Mode of inheritance | None | Unknown | Unknown |
|  | **Average number of variants in final list*** | **23** | **633** | **829** |
| **Parkinson’s disease dataset 2 - *PARK2* (R275W and M432V)** | Human Phenotype Ontology terms | None | Bradykinesia, resting tremor, postural instability, rigidity | Bradykinesia, resting tremor, postural instability, rigidity |
|  | Frequency cutoff | 0.01 | 0.01 | 0.01 |
|  | Mode of inheritance | None | Unknown | Unknown |
|  | **Average number of variants in final list*** | **2** | **621** | **820** |

*This is the average number of variants obtained across varying numbers of samples (see Table 1).
